# The impact of glucose-insulin-potassium infusion in acute myocardial infarction on infarct size and left ventricular ejection fraction [ISRCTN56720616]

**DOI:** 10.1186/1741-7015-3-9

**Published:** 2005-06-02

**Authors:** Iwan CC van der Horst, Jan Paul Ottervanger, Arnoud WJ van 't Hof, Stoffer Reiffers, Kor Miedema, Jan CA Hoorntje, Jan-Henk E Dambrink, AT Marcel Gosselink, Maarten WN Nijsten, Harry Suryapranata, Menko-Jan de Boer, Felix Zijlstra

**Affiliations:** 1 Department of Cardiology, Thoraxcenter, University Medical Center Groningen, Hanzeplein 1, 9700 RB Groningen, the Netherlands; 2 Department of Cardiology, Isala Klinieken, locatie Weezenlanden, Groot Wezenland 20, 8011 JW Zwolle, the Netherlands; 3 Department of Nuclear Medicine, Isala Klinieken, locatie Weezenlanden, Groot Wezenland 20, 8011 JW Zwolle, the Netherlands; 4 Department of Clinical Chemistry, Isala Klinieken, locatie Weezenlanden, Groot Wezenland 20, 8011 JW Zwolle, the Netherlands; 5 Department of Surgery, University Medical Center Groningen, Hanzeplein 1, 9700 RB, Groningen, the Netherlands

## Abstract

**Background:**

Favorable clinical outcomes have been observed with glucose-insulin-potassium infusion (GIK) in acute myocardial infarction (MI). The mechanisms of this beneficial effect have not been delineated clearly. GIK has metabolic, anti-inflammatory and profibrinolytic effects and it may preserve the ischemic myocardium. We sought to assess the effect of GIK infusion on infarct size and left ventricular function, as part of a randomized controlled trial.

**Methods:**

Patients (n = 940) treated for acute MI by primary percutaneous coronary intervention (PCI) were randomized to GIK infusion or no infusion. Endpoints were the creatinine kinase MB-fraction (CK-MB) and left ventricular ejection fraction (LVEF). CK-MB levels were determined 0, 2, 4, 6, 24, 48, 72 and 96 hours after admission and the LVEF was measured before discharge.

**Results:**

There were no differences between the two groups in the time course or magnitude of CK-MB release: the peak CK-MB level was 249 ± 228 U/L in the GIK group and 240 ± 200 U/L in the control group (NS). The mean LVEF was 43.7 ± 11.0 % in the GIK group and 42.4 ± 11.7% in the control group (P = 0.12). A LVEF ≤ 30% was observed in 18% in the controls and in 12% of the GIK group (P = 0.01).

**Conclusion:**

Treatment with GIK has no effect on myocardial function as determined by LVEF and by the pattern or magnitude of enzyme release. However, left ventricular function was preserved in GIK treated patients.

## Background

It has been suggested that glucose-insulin-potassium (GIK) infusion in acute myocardial infarction (MI) has clinical benefit [1-4]. Both animal studies and early studies in patients to investigate the influence of GIK on infarct size have shown conflicting results [5-14]. Experimental studies on the effect of GIK on preservation of left ventricular function, as determined by hemodynamic parameters, demonstrated a beneficial effect [15,16]. Furthermore, in a small study in patients with acute MI treated by thrombolysis, there was a significant improvement in left ventricular function over a 10-day period [17]. Recently, a small randomized trial of 37 patients has suggested that the addition of GIK to primary percutaneous coronary intervention (PCI) has a beneficial effect [18]. Large studies of GIK in the era of reperfusion yielded some information about effects on myocardial function [1,17,19]. The Polish Glucose-Insulin-Potassium (Pol-GIK) trial with 954 patients, using low-dose GIK, found no difference between median creatinine kinase (CK) levels (920 IU/L in GIK patients versus 925 IU/L in controls) [19]. Recently, in the REeValuation of Intensified Venous metabolic support for Acute infarct size Limitation (REVIVAL) trial with 312 MI patients, the combination of GIK and primary PCI had no effect on myocardial salvage [20]. In the Glucose-Insulin-Potassium Study (GIPS) with GIK infusion as adjunctive therapy to primary PCI in acute MI, the 30-day mortality was not significantly reduced in the overall population [4]. In a predefined subgroup of patients without heart-failure at admission, mortality was 1.2% in GIK patients versus 4.2% in controls (P = 0.01). In the present study we were able to determine the effect of GIK on cumulative enzyme release and left ventricular ejection fraction.

## Methods

### Study population

An outline of the study and 30-day clinical follow-up has been reported [4]. All consecutive patients with symptoms consistent with acute MI of >30 minutes duration, presenting within 24 hours after the onset of symptoms and with ST-segment elevation more than 1 mm (0.1 mV) in two or more contiguous leads on the electrocardiogram, were evaluated for inclusion in this study. Patients were excluded if they had been pre-treated by thrombolysis or when an illness associated with markedly restricted life expectancy was present. Before randomization, age, gender, history of coronary artery bypass grafting (CABG), previous PCI, stroke and MI, existence of diabetes mellitus, smoking status, heart rate, arterial pressure, body mass index, Killip class, electrocardiographic site of infarction, time of onset of symptoms, and time of hospital admission in both the referring hospital and our hospital were recorded. The research protocol was reviewed and approved by the medical ethics committee of our hospital, and patients were included after informed consent.

### Treatment protocol

Patients were randomly assigned to either GIK infusion or no infusion. Assignments to the treatment groups were made with the use of a computerized randomization program. In the GIK group, a continuous infusion of 80 mmol potassium chloride in 500 mL 20% glucose was given at a rate of 3 mL/kg/hour through a peripheral venous line. A continuous infusion of short-acting insulin (50 units Actrapid HM-Novo Nordisk, Copenhagen, Denmark) in 50 ml 0.9% sodium chloride was also initiated using a pump (Perfusor-FM, B. Braun, Melsungen, Germany). The baseline insulin-infusion dose and hourly adjustments of the dose to obtain blood-glucose levels between 7.0 and 11.0 mmol/l were determined using an algorithm based on measurements of whole-blood glucose concentration. The infusion was started as soon as possible after randomization and continued for at least 8 hours with a maximum of 12 hours.

All patients went to the catheterization laboratory as soon as possible after admission and randomization. In the laboratory, both coronary arteries were visualized. PCI was performed using standard techniques if the coronary anatomy was suitable for angioplasty. Additional treatment consisted of unfractionated intravenous heparin, nitroglycerin and aspirin. After sheath removal, low-molecular-weight heparin was given for 1 to 3 days. Before discharge, additional pharmacological treatment consisting of aspirin, clopidogrel, coumarins, beta-blockers, angiotensin-converting enzyme inhibitors and statins was initiated according to the guidelines of the American College of Cardiology/American Heart Association. Enrollment of patients began in April 1998 and ended in September 2001.

### Measurements

Enzymatic infarct size was estimated by serial measurements of the CK-MB fraction. The first measurement was taken as soon as possible after admission. Thereafter frequent CK-determinations were performed according to a schedule that called for 4 to 8 measurements in the first 96 hours. To accommodate the problem that exact predefined times for blood sampling were not always followed in practice, the actual times of sampling were recorded, expressed as minutes after the moment of randomization. To allow optimal comparison of the time courses of CK-MB levels and to best approximate the area under the CK-MB curves, we developed an algorithm. This algorithm estimated the time-course of CK-MB for each patient before calculating the patient group means. According to the known kinetics of CK-MB – a rapid rise and a much slower decrease – the following characteristic time points with accompanying time intervals were defined: 0 h (interval from 60 min before to 45 min after randomisation); 2 h (45 min to 3 h); 4 h (3 h to 5 h); 6 h (5 h to 12 h); 24 h (12 h to 36 h); 48 h (36 h to 60 h); 72 h (60 h to 84 h) and 96 h (84 h to 108 h). The algorithm interpolated the CK-MB level for each patient based on the precise times at which the measurements were performed. Next, it determined for each of the predefined intervals whether an actual measurement had been performed in that interval. If no measurement was available for a particular interval, a "not available" value was generated. This avoided inappropriately interpolated values. If one or more measurements were available for an interval, the CK-MB value for the time point with the corresponding interval was calculated from the area under the interpolated curve divided by the interval duration. CK-MB was determined enzymatically using a Hitachi 717 automatic analyzer at 30 degrees Celsius according to the International Federation of Clinical Chemistry (IFCC) recommendation. High enzyme release was defined as a peak CK-MB above the 75% percentile (i.e. the highest quartile). Time to peak CK-MB was determined.

Left ventricular ejection fraction (LVEF) was measured before discharge by radionuclide ventriculography or by echocardiography. Radionuclide ventriculography was performed by the multiple gated equilibrium method following the labeling of the patient's red blood cells with technetium 99mpertechnate. A General Electric 300 gamma camera with a low-energy all-purpose parallel-hole collimator was used. The global ejection fraction was calculated with a Star View computer (General Electric, Wisconsin, USA) using the fully automatic PAGE program. LVEF as assessed by two-dimensional transthoracicechocardiography was reported as a descriptive grade of function, using subjective visual assessment by two independent observers who were unaware of the randomisation code. This approach is less time-consuming than other methods, such as Simpson's rule or wall motion index score, but it can be at least as accurate as other methods [21]. Echocardiography was performed by observers who were unaware of the randomization outcome and clinical data. A LVEF ≤ 30% was defined as a poor left ventricular function before the analysis.

### Statistical analysis

All analyses were by intention to treat. Predefined endpoints were pattern and magnitude of CK-MB release, peak CK-MB, high enzyme release (defined as a peak enzyme release in the highest quartile); mean LVEF, and a LVEF lower than or equal to 30%. To allow comparison of the patterns of CK-MB release, the CK-MB values 0, 2, 4, 6, 24, 48, 72 and 96 hours after admission were calculated for each individual patient with linear interpolation. Differences between group means were assessed with the two-tailed Student's t-test. Chi-square analysis was used to test differences between proportions. The Cox proportional-hazards regression model was used to calculate relative risks adjusted for differences in baseline characteristics. To predict the independent association between treatment with GIK and a peak CK-MB release above the highest quartile or LVEF ≤ 30%, a multivariate logistic regression analysis was performed. A two-tailed P value <0.05 was considered statistically significant. The Statistical Package for the Social Sciences (SPSS Inc., Chicago, IL, USA) version 11.5 was used for all statistical analyses.

## Results

### Study population

Of the 940 patients who underwent randomization, 476 were assigned to receive GIK infusion. There were no differences in the baseline characteristics of the two treatment groups table 1. The mean time between onset of symptoms and admission was 150 min, and 75% of the patients were admitted within 225 min. The interquartile range (IQR) in the GIK group was 100 to 215 min and in the control group 105 to 234 min. After coronary angiography, 860 patients (90.5%) underwent PCI, 38 patients (4%) were referred for CABG within 7 days, and 42 patients (4.5%) were treated conservatively. Thirty-day mortality was 4.8% in the GIK patients and 5.8% in the control patients [4].

**Table 1:** Baseline clinical and demographic characteristics and initial therapies

Characteristics	GIK group	Control group
Age, years ± SD	60 ± 12	61 ± 12
Men	351 (73.7)	368 (79.3)
Referred patients	201 (42.3)	180 (38.8)
Body mass index	26.7 ± 3.8	27.0 ± 4.0
Previous MI	52 (10.9)	53 (11.4)
Previous PCI	22 (4.7)	24 (5.2)
Previous CABG	14 (2.9)	12 (2.6)
History of stroke	17 (3.6)	15 (3.2)
Diabetes mellitus	50 (10.5)	49 (10.6)
Hypertension	134 (28.2)	130 (28.0)
Dyslipidaemia	94 (19.7)	95 (20.5)
Currently smoker	225 (47.3)	237 (51.1)
Positive family history	195 (41.0)	179 (38.6)
Time to admission, min (IQR)*	150 (100–215)	150 (105–234)
Door to balloon time, min (IQR)	45 (31–64)	48 (34–69)
Killip class 1	426 (89.5)	430 (92.7)
Killip class 2	24 (5.0)	14 (3.0)
Killip class 3	14 (2.9)	14 (3.0)
Killip class 4	12 (2.5)	6 (1.3)
Anterior MI	250 (52.9)	224 (49.1)
Multi-vessel disease	249 (52.3)	242 (52.2)
PCI	436 (91.6)	424 (91.4)
Stent **	255 (58.5)	236 (55.7)
GP IIb/IIIa receptor blocker **	96 (22.1)	109 (25.7)
In-hospital CABG	19 (4.0)	19 (4.1)
TIMI 3 flow	459 (96.4)	435 (93.8)
Successful reperfusion‡	394 (82.8)	384 (82.8)
Intra-aortic balloon pump	45 (9.5)	42 (9.1)
Mechanical ventilation	12 (2.5)	4 (0.9)

Data are numbers (%) unless otherwise indicated. MI = myocardial infarction. IQR = interquartile range. PCI = percutaneous coronary intervention. CABG = coronary artery bypass grafting. * Time to admission denotes time between onset of symptoms and admission. † In-hospital CABG = CABG defined as patients treated initially conservatively, followed by elective CABG within 7 days. ‡ Successful reperfusion denotes TIMI 3 flow and blush grade 2 or 3. ** Percentage defined as the percentage of the patients who underwent PCI.

### Enzyme release

**Figure 1:**
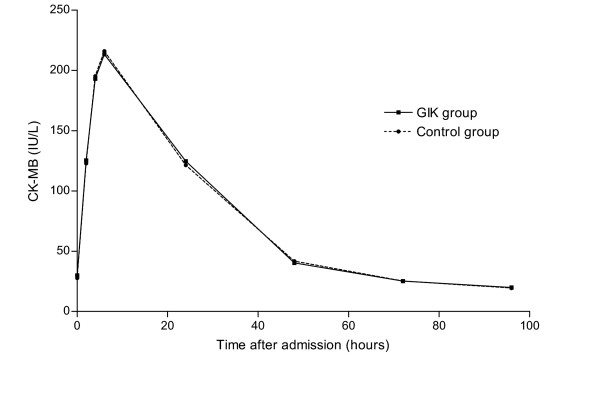
CK-MB release after admission in the GIK group and control group. CK-MB release after admission in the GIK group (unbroken line) and control group (dashed line). Means are indicated for 0, 2, 4, 6, 24, 48, 72 and 96 hours. No significant difference was observed at any time point.

Data on the effect of GIK on enzymatic infarct size (CK-MB) were available in 923 patients (98%), 470 patients treated with GIK and 462 control patients. A mean of 6.9 CK-MB determinations per patient were available. As indicated in figure 1, there was no difference in the pattern or magnitude of CK-MB release between the two groups. Mean peak CK-MB ± SD was 249 ± 228 U/L in the GIK group and 240 ± 200 U/L in the control group (NS). In GIK patients the median time to peak CK-MB was 450 min (IQR 300–733) compared to 468 min (IQR 285–738) in the control patients (NS). High enzyme release (i.e. peak CK-MB >349 U/L) was found in 111 patients (24%) in the GIK group versus 121 patients (25%) in the control group (P = 0.6). Differences between patients with peak CK-MB release below and above 349 U/L are indicated in table 2. Characteristics related to high enzyme release were Killip class ≥ 2, anterior MI and TIMI 0 flow before PCI.

**Table 2:** Comparison of patients with a peak CK-MB release beneath the highest quartile and patients with a CK-MB release above the highest quartile

Characteristic	High enzyme release (N = 232)	Low enzyme release (N = 708)	P-value
Age, years ± SD	59 ± 12	61 ± 12	0.17
Men	179 (77)	540 (76)	0.86
Hypertension	64 (28)	200 (28)	0.87
Previous MI	24 (10)	81 (11)	0.72
Diabetes mellitus	28 (12)	71 (10)	0.39
Killip class 1	192 (83)	664 (94)	<0.001
Anterior MI	161 (69)	313 (44)	<0.001
TIMI 0 before PCI	189 (82)	377 (53)	<0.001
TIMI 3 after PCI	219 (94)	675 (95)	0.6
GIK treatment	121 (52)	355 (50)	0.6

Data are numbers (%) unless otherwise indicated. MI = myocardial infarction. TIMI = Thrombolysis in Myocardial Infarction. PCI = percutaneous coronary intervention.

### Left ventricular ejection fraction

LVEF measurements were available in 419 patients (88%) treated with GIK and in 404 (87%) of the controls. Mean time between admission and LVEF measurement was 2.65 days in the GIK group and 2.5 days in the control group (IQR 2 days in both groups). The mean LVEF was 43.7 ± 11.0 % in the GIK group and 42.4 ± 11.7% in the control group (P = 0.12). Of the 823 patients with a measured LVEF before discharge, a total of 125 (15%) had an ejection fraction lower than or equal to 30%. Differences between patients with LVEF ≤ 30% and those with a higher LVEF are summarized in table 3. Treatment with GIK was associated with a lower frequency of a LVEF ≤ 30% (51 patients (12%) compared to 74 patients (18%), P = 0.01). Other important characteristics associated with LVEF ≤ 30% were age, diabetes mellitus, Killip class, infarct location and TIMI flow after PCI. To identify independent predictors of a low left ventricular ejection fraction (LVEF ≤ 30%) all variables associated with LVEF ≤ 30% in univariate analysis were used in multivariate analysis; see table 3. After adjustment, patients treated with GIK had a RR of 0.53 (95% confidence interval 0.35–0.81, P = 0.01) for LVEF ≤ 30% compared to controls. In patients with Killip class 1 at admission the mean LVEF was 44.1 ± 10.9 % in the GIK group versus 42.8 ± 11.5% in the control group (P = 0.12). In these Killip class 1 patients randomized to GIK, a LVEF ≤ 30% was less often observed (45 patients (12%) versus 65 patients (17%), P = 0.03).

**Table 3:** Comparison of patients with a low Left Ventricular Ejection Fraction (LVEF ≤ 30%) with those with preserved Left Ventricular Ejection Fraction (LVEF >30%)

Characteristic	Low LVEF (N = 125)	Preserved LVEF (N = 698)	P-value
Age, years ± SD	63 ± 12	59 ± 12	0.008
Men	98 (78)	537 (77)	0.7
Hypertension	43 (34)	182 (26)	0.05
Previous MI	18 (14)	63 (9)	0.06
Diabetes mellitus	29 (23)	57 (8)	0.008
Killip class 1	109 (87)	660 (95)	0.002
Anterior MI	115 (92)	291 (42)	<0.001
TIMI 0 before PCI	40 (32)	291 (42)	0.04
TIMI 3 after PCI	114 (91)	677 (97)	0.002
GIK treatment	51 (41)	368 (53)	0.01

Data are numbers (%) unless otherwise indicated. MI = myocardial infarction. TIMI = Thrombolysis in Myocardial Infarction. PCI = percutaneous coronary intervention.

In the overall population the mean peak CK-MB ± SD was 463 ± 318 U/L in patients with LVEF ≤ 30% versus 215 ± 166 U/L in patients with LVEF >30% (P < 0.001).

## Discussion

In this study, additional treatment with GIK in patients treated with primary PCI for acute myocardial infarction showed no decrease in enzymatic infarct size and no difference in mean LVEF between GIK and control patients was observed. However, preservation of left ventricular function as determined by a LVEF ≤ 30% was more frequently seen in GIK treated patients. One explanation for the discrepancy between the effects on poor and mean left ventricular function is that the number of patients in our analysis was not sufficient to detect an effect on mean left ventricular ejection fraction. Mean LVEF was 1.3% higher in GIK patients, so and several patients may have been prevented from LVEF ≤ 30%.

Since these results are based on an intention-to-treat analysis in a randomized controlled trial of the effects of GIK, we may have underestimated the effect on preservation of myocardial function. In the control group more patients died, and these patients had a high enzyme release and probably a severely impaired LVEF as well. Nevertheless, our data make it unlikely that GIK has a clinically relevant impact of GIK on enzyme release, but at the same time show an important reduction in the number of patients with a LVEF ≤ 30%. A LVEF ≤ 30% exposes these patients to a high risk of developing heart failure during follow-up, even after successful PCI [22]. We also found a relation between high enzyme release and poor left ventricular function. Possibly the relation between GIK and poor LVEF is related to a selection bias in this sub-analysis. On the other hand it can be hypothesized that GIK mediates effects on LVEF, as measured after 2 to 4 days after admission, that are unrelated to enzymatic infarct size.

In our previous study on the effect of GIK on 30-day mortality, we found a beneficial effect of GIK on outcome in patients who had no signs of heart-failure at admission. Patients with Killip class 1 did not have smaller enzymatic infarct sizes in this analysis. However, the CK-MB curve over the first 96 hours after admission in GIK patients lies below that in controls. Mean LVEF is higher in patients treated with GIK compared to control patients, albeit not significantly (P = 0.12). **Even as in the overall population in patients with Killip class 1 treatment with GIK was related with a lower rate of LVEF ≤ 30%.**

### Previous clinical studies with GIK

Several studies have reported enzymatic infarct size in MI patients treated with GIK, mainly in patients without reperfusion therapy [12,14]. Clinical studies in the era of reperfusion provided sparse information [1,19]. In line with our results, the Pol-GIK trial showed that neither the median and maximal CK nor maximal glutamic-oxaloacetic transaminase differed significantly between GIK and control patients [19]. However, the Pol-GIK trial used low-dose GIK and included patients with a lower risk, i.e. no patients with signs of heart failure were included. One study of 32 MI patients treated by thrombolysis found that infusion of GIK resulted in a shorter time to peak CK and a smaller total enzyme release [6]. In line with our results on enzyme release, the REVIVAL trial found no effect of GIK treatment on the myocardial salvage index measured with **technetium-99m sestamibi scintigraphy **6 to 8 hours after admission and after 7 to 14 days (0.50 versus 0.48, P = 0.96). In this trial, GIK consisted of 1000 ml 20% glucose with 40 IU of insulin and 64 mmol potassium chloride at a rate of 1.8 ml/hour for 24 hours. However, in the predefined subgroup of patients with diabetes mellitus, a significant difference in salvage index was found (mean difference 0.19, 95% confidence interval 0.01–0.37). In a trial of 37 patients treated with primary PCI, a beneficial effect of GIK on myocardial function was observed [18]. The increase of the LVEF from baseline to 3 months was 12.5% in the GIK group (P = 0.002) versus 6.1% in the placebo group (NS). However, when GIK and placebo were compared directly, the difference between increments in LVEF was not significant. This may have been the result of a combination of small numbers of patients and profound changes in the LVEF during the 3–6 months after PCI for acute MI [23]. In patients with diabetes mellitus and without myocardial ischemia, infusion of insulin and glucose increased the contractile force, i.e. rest LVEF and LVEF during dynamic exercise measured by radionuclide ventriculography [24]. In 32 anterior MI patients, the left ventricular wall motion score index as measured by echocardiography at baseline was 1.87 and it decreased significantly during GIK infusion to 1.76 (P < 0.001) [25].

In January 2005, the 30-day results of the Clinical Trial of Reviparin and Metabolic Modulation in Acute Myocardial Infarction Treatment Evaluation-Estudios Cardiologicos Latinoamerica (CREATE-ECLA) trial (N = 20201) were published [26]. Infusion of high-dose GIK had no beneficial effect on the overall population, with a mortality rate of 10.0% in the GIK group compared to 9.7% in the control group (P = 0.45). The lack of benefit was also observed in several large subgroups that may benefit in particular from GIK, such as patients treated within 4 hours after symptom onset (N = 8361). Only in patients treated by PCI (N = 1838) was a reduction of 30-day mortality observed, from 6.3% in control patients to 4.8% in GIK patients; but this was not significant. Therefore, in patients treated with a combination of GIK and reperfusion, as in our study, the role of metabolic modulation in mortality and myocardial function needs further investigation.

### Animal studies

Several studies on animals have assessed the effects of GIK on infarct size in myocardial infarction, but show conflicting results [5,9,11]. A study of pigs showed that hearts treated with GIK had significantly less tissue acidosis, higher wall motion scores and less tissue necrosis [16]. Similar results were obtained in a study of rats, in which the administration of GIK not only before induction of ischemia but also during acute ischemia seemed beneficial for myocardial function [10]. A recent study of diabetic sheep found that GIK improved left ventricular contractility as determined by stroke work efficiency [27].

### Mechanisms of action

We found a relationship between higher enzymatic infarct size and low LVEF. Since we observed an effect of GIK only on LVEF, the preservation of left ventricular function could not be declared a reduction of myocardial necrosis. The potential mechanisms by which GIK might be beneficial include metabolic effects, direct hemodynamic effects, improvement of coronary flow and catecholamine-mediated effects. The main effect of GIK infusion is supposed to be improved delivery of glucose to the ischemic myocardium resulting in a suppression of free fatty acids [28-31]. Treatment with glucose and insulin inhibits lipolysis, thereby decreasing circulating free fatty acid levels, and suppresses fatty acid oxidation. Moreover, in the postischemic myocardium, contractile dysfunction may also be caused by impairment of glucose oxidation and cytosolic proton accumulation, so agents that enhance glucose oxidation in the postischemic heart may also improve contractile function [32]. After prolonged periods of low-flow ischemia, functional recovery is mainly determined by the extent of irreversible ischemic damage. After brief episodes of ischemic damage, oxidative metabolism rapidly returns, well before contractile activity is restored. Therefore GIK treatment is likely to be beneficial when started soon after the onset of ischemia. Protection of ischemic cardiac cell membranes may also improve reflow after reperfusion and protect against the no-reflow phenomenon by reducing cell swelling and microvascular compression [33]. These microvascular protective effects on the coronary circulation are probably similar in patients with and without diabetes mellitus [34,35]. GIK infusion improved regional myocardial perfusion and function in segments adjacent to the infarct area in patients recovering from an acute MI [36]. Another beneficial mechanism may be the protective effect of GIK on the cell membrane [37-39]. Insulin promotes tolerance against ischemic cell death via the activation of innate cell-survival pathways in the heart [40]. However, in a small study of patients with MI treated with PCI, administration of GIK did not improve the enzymatic antioxidant reserve [41].

### Study limitations

Left ventricular function was measured in 88% of patients. For some patients transferred back to the referring hospitals within 48 hours the measurement was missing. The same goes for patients who died within 48 hours. Therefore the exact relation between LVEF and 30-day mortality cannot be determined. It is unknown whether these missing data have influenced the results. We determined the left ventricular function before discharge and it has been suggested that measurements after long-term follow-up are more predictive[18,23].

## Conclusion

High-dose GIK infusion in patients with acute MI treated with primary PCI had no effect on general left ventricular function or the pattern and magnitude of enzyme release. A lower rate of poor left ventricular function, defined as a left ventricular ejection fraction ≤ 30%, was observed in GIK patients.

## Abbreviations

GIK glucose-insulin-potassium

MI myocardial infarction

PCI primary coronary intervention

CABG coronary artery bypass grafting

CK creatine kinase

LVEF left ventricular ejection fraction

TIMI thrombolysis in myocardial infarction

IQR interquartile range

RR relative risk

Pol-GIK Polish Glucose-Insulin-Potassium trial

REVIVAL REeValuation of Intensified Venous metabolic support for Acute infarct size Limitation

GIPS Glucose-Insulin-Potassium Study

CREATE-ECLA Clinical Trial of Reviparin and Metabolic Modulation in Acute Myocardial Infarction Treatment Evaluation-Estudios Cardiologicos Latinoamerica

## Competing interests

The author(s) declare that they have no competing interests.

## Authors' contributions

IH, JO, and FZ participated in the design of the study. AH, JH, AG, JD, HS, MB and FZ were responsible for patient inclusions. KM was responsible for the laboratory measurements. SR was responsible for the nuclear measurements. IH, JO, and MN were responsible for the statistical analysis. All authors have been involved in drafting the article. IH, MN, and FZ have been involved in revising the article critically. All authors have given final approval of the version to be published.

**Figure 2:**
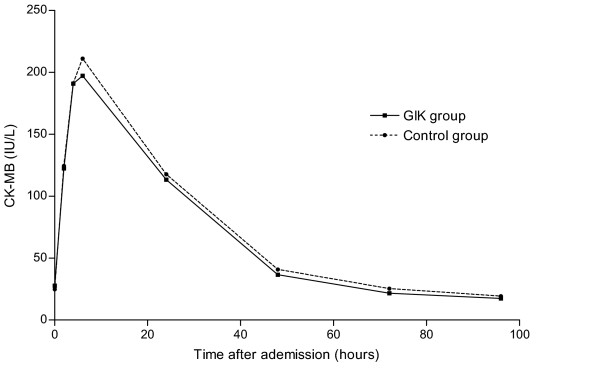
CK-MB release after admission in the GIK group and control group of Killip class 1 patients. CK-MB release after admission in the GIK group (unbroken line) and control group (dashed line). Means are indicated for 0, 2, 4, 6, 24, 48, 72 and 96 hours. No significant difference was observed at any time point.

## Pre-publication history

The pre-publication history for this paper can be accessed here:


